# Diagnosis of *Helicobacter pylori*: Changes towards the Future

**DOI:** 10.3390/diseases3030122

**Published:** 2015-06-29

**Authors:** Behnam Kalali, Luca Formichella, Markus Gerhard

**Affiliations:** 1 Institute of Medical Microbiology, Immunology and Hygiene, TU-Munich, Troger Str.30, 81675 Munich, Germany; E-Mails: behnam.kalali@tum.de (B.K.); luca.formichella@tum.de (L.F.); 2 ImevaX GmbH, Grillparzer Str.18, 81675 Munich, Germany; 3 German Center for Infection Research (DZIF), Partner site Munich, Troger Str.30, 81675 Munich, Germany

**Keywords:** *Helicobacter pylori*, diagnosis, invasive methods, non-invasive methods

## Abstract

Since the first evidence demonstrating the dramatically high incidence of *H. pylori* infection and the subsequent medical challenges it incurs, health management of *H. pylori* infection has been a high priority for health authorities worldwide. Despite a decreasing rate of infection in western countries, prevalence of *H. pylori* infection in developing and in some industrial countries is still very high. Whereas treatment and vaccination against *H. pylori* is a contemporary issue in medical communities, selective treatment and prior high-throughput screening of the subject population is a major concern of health organizations. So far, diagnostic tests are either elaborative and require relatively advanced medical care infrastructure or they do not fulfill the criteria recommended by the Maastricht IV/Florence consensus report. In this review, in light of recent scientific studies, we highlight current and possible future approaches for the diagnosis of *H. pylori*. We point out that novel non-invasive tests may not only cover the requirements of gold standard methods in *H. pylori* detection but also offer the potential for risk stratification of infection in a high throughput manner.

## 1. Introduction

*Helicobacter pylori* is a gram negative microaerophilic fastidious bacterium which over centuries has successfully infected around 50 percent of human individuals throughout the world. Very often, infection occurs in childhood and persists lifelong if not treated. This human pathogen is known to induce several gastric disorders, but may also be associated with extragastric diseases like anaemia, dyspepsia, and some immunological disorders [1,2,3]. Almost all infected subjects develop chronic gastritis, and a considerable percentage of patients further develop ulcer disease or gastric cancer. Direct and indirect evidence suggests that the eradication of *H. pylori* or vaccination against it may decrease the risk of ulcer disease and gastric cancer. On the other hand, this approach is questioned by several scientists arguing that the co-evolution of *H. pylori* with the human population might imply beneficial effects of this bacterium for its host. Indeed, *H. pylori* infection has been shown to protect from childhood diarrhoea and asthma [4,5]. Here, the chronic infection leads to a constant stimulation of the immune system, the consequences of which might be more than local inflammation; it might lead to a persistent status of activation and induce a shift in immune regulation which could influence the course of many other diseases including asthma [6]. Therefore, prior to any treatment of *H. pylori* infection especially in larger populations and in asymptomatic individuals, exact diagnosis of the infection and characterization of the bacterium in the context of pathogenesis and virulence is necessary [7,8].

Soon after the initial discovery of *H. pylori* by Marshall and Warren in 1983 and the subsequent confirmation of its causal role in gastric diseases, means of correct diagnosis as well as the clinical management of infected patients became a challenge to medicine and related biological sciences. Despite numerous academic research projects and commercial approaches, a standard method uniformly applicable, especially for indigent risk populations, is still missing. This, together with the high infection incidence worldwide on the one hand and the increasing need for individualized treatment of this infection on the other, demonstrates the emerging need of highly competent diagnostic methods for *H. pylori* [9,10]. While these methods should fulfill the common standards of clinical diagnostics like accuracy, sensitivity, and specificity, they should also be applicable in developing countries (areas) where hygiene standards and medical support are low. Certainly, the costs, time, necessary equipment and human resources as well as the availability of point of care and high throughput application are important issues which must be considered in the development of such a method. Currently, there are increasing numbers of attempts for development and several routinely employed methods for the diagnosis of *H. pylori*. According to the guideline of the Maastricht IV/Florence consensus report for the management of *H. pylori* infection, while the majority of older but still gold standard methods belong to the invasive approaches, non-invasive methods are more desirable and could cover the requirements for the more suitable tests as described above more conveniently [11]. In this review we attempt to categorize these tests, briefly describe their advantages and disadvantages and finally provide some hints regarding a future oriented standard test.

## 2. Invasive Methods

### 2.1. Rapid Urease Test

The Rapid Urease Test (RUT) is a popular invasive diagnostic *H. pylori* test that is relatively quick, cheap and simple to perform. It detects the presence of urease in or on the gastric mucosa. Best results for RUT are obtained if biopsies are taken from both the antrum and corpus. The biopsy used for RUT can also be used for other tests such as for molecular-based tests of microbial susceptibility or for host factors [12]. False positive results are rarely observed. However, treatment with proton pump inhibitors or antimicrobial agents, which prevent the growth of urease producing *H. pylori* prior to the RUT, may cause false negative results. Moreover, the sensitivity of the test in patients with peptic ulcer disease with bleeding as well as in patients with intestinal metaplasia is significantly lower [12,13]. Therefore in these cases a negative RUT result should be complemented by a second method.

### 2.2. Histology

The presence of typical spiral motile bacteria accompanied by inflammatory reaction in the histo-pathological sections of stomach was the first described method used for the diagnosis of the *H. pylori* [14]. Along with routinely applied stainings like Giemsa, hematoxylin, and eosin, there are some more specific staining procedures which facilitate the diagnosis of *H. pylori* infection. However, the accuracy of the histo-pathological diagnosis of *H. pylori* always depends on the number and the location of collected biopsy materials. While *H. pylori* can be detected in even a single biopsy taken from the correct site, to achieve a higher sensitivity, multiple biopsies are recommended. Moreover, the possible presence of other bacterial species with a similar morphology to *H. pylori* in the stomach [15,16] can be another source of error which negatively affects the accuracy of the test. In addition, treatment with proton pump inhibitors (PPI) or antibiotics prior to sampling may transform the shape of *H. pylori* to a coccoid form. Although molecular biology techniques like *in situ* hybridization [15] or immunological methods [17] are helpful solutions for the pitfalls mentioned above, the long assay time of between three hours and three days and the requirement of a relatively complex infrastructure for these methods compromise routine application.

### 2.3. Culture

Although it should be stated that *H. pylori* culture is not a routine procedure in initial diagnosis, in many bacteriology laboratories *H. pylori* isolation via the culture of biopsy samples is a routine second line approach [18,19]. Because of the demanding character of this bacterium, this method remains challenging. This technique, although highly specific, is not as sensitive as other tests like histology and the rapid urease test. As well as for purposes of scientific research, cultured live *H. pylori* is used for diagnostic approaches and for the detection of antibiotic resistance if treatment failure is suspected [20]. *H. pylori* requires a microaerophilic atmosphere (5% to 10% oxygen, 5% to 12% carbon dioxide and 80%–90% nitrogen with humidity) and a complex culture media. The most commonly used media contains Brucella, Columbia Wilkins-Chalgren, brain-heart infusion or trypticase agar bases, supplemented with sheep or horse blood [21]. Because isolation of this microaerophilic organism from gastric biopsy specimens takes a long time, up to 5–7 days, to overcome the problem of growth of other competitors that exist in the sample, the culture media is supplemented with specific antibiotics. Although *H. pylori* could be cultured from stool specimens [22,23,24] due to the presence and growth of numerous other bacteria and especially microorganisms phenotypically similar to *H. pylori*, colonies have to be further characterized by other methods. Moreover, it is possible that the bacterium goes into a viable form that cannot be cultured (coccoid form) which leads to false negative results [25].

### 2.4. PCR

PCR based detection of *H. pylori* could be categorized under invasive as well as non-invasive methods. Molecular diagnostics have dramatically changed the clinical management of many infectious diseases in the past decades. PCR currently remains the best developed molecular technique as it provides a wide range of clinical applications, including specific or broad-spectrum pathogen detection, evaluation of emerging novel infections, surveillance, early detection of bio threat agents, and antimicrobial resistance profiling [26]. While PCR could be applied for the detection of *H. pylori* in biopsies, this technique is more qualified for its use in samples taken from the oral cavity or from stool. PCR-based techniques, if applied as a non-invasive approach (*i.e.*, from stool samples) tend to be more cost effective than other traditional methods. In addition to the improved specifications of this technology like high sensitivity and specificity, simplicity, and automated procedures, there are several other advantages to be considered. Practically, regardless of genome size, any genomic material could be used as a template sample for PCR, which allows sampling from multiple origins [27]. The high efficiency of this method also achieves fast results. Since antibiotic resistance is currently the major challenge in microbiology, it has to be pointed out that the fast acquirement of results of not only the diagnosis of *H. pylori* but also of its susceptibility to the right antibiotics is extremely important. There are a couple of PCR methods like multiplex PCR which have been employed and recommended as an alternative to culture for resistance testing to antibiotics like clarithromycin [28,29,30,31]. However, a limitation lays in the fact that PCR cannot detect metronidazole resistance. Here, bacterial culture would still be necessary to cover this pitfall especially if a resistance to antibiotics is suspected or again in the case of second line therapies.

The achievement of the criteria mentioned above with its advantages is possible through different PCR approaches. In addition to the conventional PCR methods, a new approach like the colorimetric detection of *H. pylori* DNA using isothermal helicase-dependent amplification is a valuable tool. The rapid application of the test is complemented by reasonably high (up to 95%) sensitivity and specificity of the assay [32].

Despite the numerous advantages of PCR-based techniques, there are still some challenges regarding its appropriate application in diagnostics. While correct sampling and preparation of samples prior to assay is a common issue in diagnostic assay, sensitivity and specificity of PCR-based assay are strongly dependent on the design of the method. For instance, due to the extraordinary variability of *H. pylori* genome, the selection of the target gene and PCR primer pairs dramatically influence specificity and sensitivity of the test [33]. As the whole genome of most known pathogens including many *H. pylori* strains has been successfully sequenced in the last decade, it is very important to design and select the PCR primers based on a comprehensive bioinformatics analysis of relevant genomes. Here, it is mandatory to design primers according to genomic sequences that are highly conserved in all *H. pylori* strains.

## 3. Non-Invasive Methods

### 3.1. Urease Breath Test

The urease breath test (UBT) is one of the most common non-invasive tests used. This non-invasive test, available in different versions, has been evaluated in different studies, showing high sensitivity, specificity and accuracy [34,35]. The test is able to detect the infection indirectly by measuring the existence of bacterial urease produced by *H. pylori* in the stomach. There are different types of this test comprising 13C- or 14C-isotope labelled urea. If *H. pylori* is present, the urease hydrolyses the labelled urea and the exhaled isotope containing ammonia can be detected applying the samples to a measuring device. This test is recommended by the Maastricht IV/Florence Consensus Report as a valuable diagnostic tool for the detection of infection and for therapy control [11].

It has been shown that UBT can distinguish an ongoing from a past infection; hence, it is able to detect the eradication progress after treatment [36]. A recent study was conducted comparing C13-UBT and a monoclonal *H. pylori* stool antigen test in infants and toddlers in South America [37]. The study suggests that both tests are reliable for *H. pylori* diagnosis in very young patients because of the high concordance between test results. In comparison, a study performed in Turkey evaluating C14-UBT in elderly patients also showed good diagnostic parameters with a sensitivity and specificity of 91.4% and 93.8%, respectively, compared to histopathology [38].

In recent years, different application protocols and detection devices were developed. A recently published study describes a residual gas analyser-based mass spectrometry approach with possible point-of-care application [39]. A further study has evaluated a low dose capsule-based UBT approach compared to conventional UBT and invasive tests [40]. This approach seems to be superior to the conventional UBT in terms of sensitivity and specificity, resulting in 100% sensitivity and specificity but depending on the time of sample collection.

One of the limitations is the presence of other urease producing bacteria in the stomach, e.g. *H. heilmannii*, which might lead to false positive results. Moreover, acute bleeding and co-medication can lead to false negative test results [41]. For reasons of required fasting prior to taking the test, it might be onerous for the patient. In addition, because of the detection devices needed, costs tend to be higher compared to alternative tests used [42]. Furthermore, according to different protocols available, the accuracy of UBT test results depends on the amount of urea applied, the point in time in which samples are taken and the set point of the cut off value [43].

### 3.2. Fecal Antigen Test

Fecal antigen tests detect antigens in stool samples. ELISA formats comprising monoclonal antibodies against *H. pylori* proteins showed improved results compared to polyclonal approaches [44]. The current guideline evaluates the use of the stool antigen test as equivalent to the UBT if a validated laboratory-based monoclonal antibody is used [11]. Korkmaz *et al.* compared the diagnostic accuracy of five different stool antigen tests in adult dyspeptic patients comparing monoclonal enzyme immunoassay tests (EIA) and rapid immunochromatographic assays (ICA). The sensitivity and specificity of the tests analysed had a high variation between 48.9%–92.2% and 88.9%–94.4%, respectively, depending on the test format. They conclude that EIA tests are more accurate compared to the currently available ICA-based test, that are fast and easy to use but provide less reliable results [45]. A recent meta-analysis conducted by Zhou and colleagues analysed forty-five studies, including 5931 patients and evaluated the test performance of a *H. pylori* stool antigen test in children. The average sensitivity and specificity was 92.1% and 94.1%, respectively [46]. Furthermore, the available stool antigen tests have been shown to be able to distinguish infected from treated patients [47], enabling the confirmation of treatment. Degradation of antigens in the intestine and consequent disintegration of epitopes might lead to false negative results. Moreover, the process of sample handling could be fastidious for patients. False negative results may occur when the bacterial load is low, due to proton-pump inhibitors or the recent use of antibiotics or bismuth [41,48].

### 3.3. Serological Test

Immune responses against *H. pylori* are utilized to detect infection by analysing patients’ blood or serum for IgG and IgA antibodies. Serology is the only test which is not affected by those local changes in the stomach that could lead to a low bacterial load and to false negative results [11]. According to guidelines proposed by the Maastricht conference, only IgG detection is considered and the favoured method is ELISA. Currently, different formats of serological tests are available, including simple ELISAs that use whole lysates or recombinant produced *H. pylori* proteins as antigens. More recently, immuno-blots [49], luminex-based bead assays [50] and line assays [51] were developed; these allow a more specific evaluation of the infecting *H. pylori* strain in terms of bacterial virulence factors and host immune responses towards the human pathogen. Moreover, they show improved sensitivity and specificity [51,52,53] because additional and highly purified antigens are included. These non-invasive tests are easy and cheap to perform. The potential for developing a rapid diagnostic test makes serology an interesting option for testing populations in areas with little or no access to medical facilities. Using an automated approach, large cohorts could be tested within a short time, allowing population based studies. Good diagnostic parameters in terms of robustness, sensitivity and specificity are given as shown in different studies [51,52]. These studies show that improved serological test formats lead to increased sensitivity of over 95% and specificity ranging from 85%–96%, making serology a preferable alternative to other non-invasive tests. However, there are also contradictory studies with low sensitivity and specificity values for the serological test [54], probably due to the test format applied or the antigens used.

Risk stratification might be an interesting issue, detecting combined antibody responses to different virulence factors. Promising studies have analysed different *H. pylori* antigens, showing significant correlations between positive immune responses and clinical outcomes like chronic atrophic gastritis, intestinal metaplasia, dysplasia, and gastric cancer [55,56,57]. In particular, there have been great improvements by applying single defined recombinant *H. pylori* antigens; the well-characterised oncogenic protein CagA that has been shown to correlate with severe gastric disorders and VacA that are in clinical use [58,59]. The immune responses towards VacA could be even more specific by differentiating between m1 and m2 variants of VacA [60]. More recently, antigens like NapA, GroEL, HyuA have been shown to have the potential to be used as biomarkers to predict infected individuals having a higher risk of developing premalignant changes that are a hallmark of gastric cancer development [61]. The combination and evaluation of different biomarkers adapted to the region where they are used might be the future method for ruling out patients at higher risk of developing severe diseases.

Antibody responses towards antigens are sustained for a long period after eradication therapy, as shown in different studies [62,63,64,65]. These studies argue that a confirmation of treatment success by serology is not applicable for this reason.

However, while some immune responses to certain antigens persist for a long period of time (especially CagA), for some antigens the antibody titre decreases within a short period, also depending on the Ig-class tested [66]. This phenomenon could also be utilized as clinical readout to confirm treatment success by analysing the decline of antibody responses, as shown in different studies [67]. Wang *et al.* conclude from their study that it would be the reasonable and even perhaps preferred method of monitoring *H. pylori* infections [68].

### 3.4. Rapid Diagnostic Test

Despite numerous publications on rapid diagnostic testing (RDT) methodologies for infectious diseases, such testing has become neither commonplace nor an integral component of services offered by clinical microbiology laboratories [69]. However, because of the emerging need for rapid diagnosis and treatment of virulent strains of different viral and bacterial infections, discussions regarding routine application of RDT are increasing within current medical circles. Debate on the value of RDT has broadened and continues to increasingly encompass new infections.

Results of RDT testing become available within a couple of minutes to a few hours. A clinical specimen is processed in a few steps (preferably in a single step) at the site where it is collected (point of care). The quality and value of the RDT is determined by its sensitivity and specificity, the time required for results, and its cost and availability. According to the guidelines set forth by the Maastricht conference, it appears that those antigens with either high or low molecular weight are more specific [11].

There are currently many *H. pylori* RDT kits commercially available. However, how far these tests fit in with standard clinical practice is still undetermined.

As generally in microbiology laboratories, *H. pylori* RDT kits could also be performed based on the following four approaches: (A) testing of *H. pylori* specific antigens; (B) molecular detection of the specific *H. pylori* nucleic acid sequence; (C) rapid biochemical reaction test and (D) serologic detection of *H. pylori* specific antibodies.

Regardless of which approach is applied, these tests have to be validated based on gold standards. In addition to specificity and sensitivity, the value of the overall agreement with the gold standard (biopsy or culture) plays a crucial role in the evaluation of the kit. Although the majority of the approved RDTs for *H. pylori* reach a non-ideal sensitivity and specificity (in general < 90%), there are promising findings that lead to significant improvement of the assays. For instance, in the process of testing of *H. pylori* specific antigens, *H. pylori* specific antibodies are required. While antibodies produced by immunization with whole *H. pylori* lysate or a mixture of bacterial compartments are more prone to be sensitive, they reduce the specificity of the test. On the other hand, antibodies which are produced against single molecules are very specific, although they gain more false negative results. The same argument is also valid in other sero-immunology based approaches that aim at detecting *H. pylori* specific antibodies. While utilization of single conserved molecule of *H. pylori* leads to reasonable specificity, this assay may lead to higher false negative results compared to the application of a mixture of antigens, which in turn may cause higher false positive results.

As mentioned above, new promising studies have described new *H. pylori* antigens which have been successfully tested in preliminary assays. For example, FliD, a hook flagellar protein of *H. pylori*, is a highly conserved molecule in all *H. pylori* species. While this molecule has no or only low homology to other bacterial species which prevents cross reactivity, it has shown a strong antigenicity in animal studies. Assays which employ this protein as antigen in the diagnosis of anti *H. pylori* antibodies have shown high sensitivity and specificity [70].

Serologic tests should therefore include antigens for sensitivity, specificity testing, and antigens that identify infections by more pathogenic *H. pylori* strains, which might provide the basis for decisions about further treatment.

## 4. Discussion

Continuous developments in both invasive and non-invasive based methods for detection of *H. pylori* infection will greatly contribute to further improvement of the health management of *H. pylori* associated disorders. Although this mysterious bacterium has been unanimously declared a human pathogen, only a minority of infected individuals develop an associated disease, which seems mainly attributed to the armament of virulence factors of *H. pylori*. Therefore, individual identification of virulence factors of the bacterium for risk stratification is discussed for risk assessment in combination with histopathological evaluation of gastritis. There are common diagnostic methods for detection of the infection and verification of the virulence factors. However, while gold standard methods are still mainly derived from biopsy-based methods, the high prevalence of the infection especially in areas with low medical support suggests the urgent need for introducing non-invasive and preferably high throughput applicable procedures. The selection of such a test depends on the sensitivity, specificity, availability, complexity, costs, and rapidity of results [71]. Unfortunately, none of the currently used methods cover these criteria perfectly. Although biopsy-based methods have a very high specificity, only a moderate sensitivity is observed in these assays mainly due to factors that are difficult to control such as the sampling site. Even in spite of correct sampling, there are other factors such as PPI treatment prior to sampling that possibly affect the growth or presence of detectable curved bacteria. PCR-based tests have slight advantages compared to other tests [71,72]. PCR, as a highly sensitive and specific method, is applicable not only in the detection of infection with *H. pylori* but also in the monitoring of treatment and therapy efficiency. Our experience showed that with the proper design of PCR, a single copy of genomic DNA is sufficient to obtain a positive signal indicating the presence of *H. pylori* in the sample. Moreover, DNA samples isolated from different sources such as gastric biopsy, samples from the oral cavity, and stool specimens could be subjected to PCR assay. Accordingly, precluding the possible chances of contamination, PCR could be used as a gold standard. The main disadvantage of PCR in our opinion is the level of diagnostic facilities and implementation of this method in regions with poor medical support. Since other biopsy-based methods generally have disadvantages such as the requirement of endoscopic facilities or additional systems such as RUT, they are not altogether suitable as gold standards. Recently, most efforts to introduce an adequate gold standard are focused on non-invasive methods. In addition to PCR, serological methods aiming at the detection of *H. pylori* specific antigens in different specimens have been significantly improved. Bioinformatics analysis greatly helps the selection of the optimal antigen applied in serological assays. Not only could the antigenicity of the antigen be predicted in these analyses, more importantly the homology of the antigen to other relevant microorganisms can be comprehensively analysed. Through the choice of a suitable antigen and the application of advanced methods in nanotechnology in assay design, new serological tests have recently reached relatively ideal performance [70]. Although the report of the Maastricht conference emphasizes that antibodies against *H. pylori* and especially against its most specific antigen CagA, remain elevated for long periods, for months or even years, there is evidence that while CagA is a dominant virulence marker, it is only present in around 70% of *H. pylori* strains depending on their geographic origin [51,73,74]. Here, recent studies identified other antigens in all *H. pylori* strains which possess not only high immunogenicity but also elicit specific antibodies which will fade away after a relatively short time after eradication. Advanced and simplified specific antibody detection techniques like line assay and rapid diagnostic tests have greatly improved the application of serological tests for the detection of *H. pylori*. These approaches cover most of the criteria mentioned for *H. pylori* diagnostic assays. Not only are they applicable in high-through-put studies of large cohorts but they are also highly cost effective, bedside applicable, simple for analysis and intelligible for medical personnel. Less so, but still in an equally acceptable relevant context, specific antigen tracing tests are currently being developed. In particular, noticeable progression has been made in the area of stool tests. Indeed, when a study focuses particularly on children of a high prevalence area, antigen-tracing systems like the stool antigen test are advantageous. Finally, there is apparent evidence suggesting that not every strain of *H. pylori* is harmful and should be treated [8,75,76,77]. In addition, there are accumulating data on the beneficial aspects of infection with *H. pylori* for its human host [75]. Therefore, conduction of risk stratification according to the epidemiological data is highly recommended. The feasibility of such assessments is now exclusively conceivable through serological assays, which can indicate the infection with pathogenic strains of *H.* *pylori*.

In conclusion, we feel that current available diagnostic systems cannot meet the requirements posed by such a widespread and prevalent infection as *H. pylori*, especially when more than simple detection of positivity is required (*i.e.* risk stratification, antibiotic resistance). We thus suggest a collaborative effort from experts and health organizations across the world to strive for the development, validation, and introduction of such multi-tasking diagnostic tools which could not only become a new recommended gold standard for the screening of large infected populations but would also assist in guiding the treatment strategies for each infected individual.
